# Supplementary material for: Analyzing Atomic Interactions in Molecules as Learned by Neural Networks
Source: J Chem Theory Comput. 2025 Jan 10;21(2):714–29. doi: 10.1021/acs.jctc.4c01424 (PMC11780731; doi:10.1021/acs.jctc.4c01424)
Supplement: Supplementary file 1 — ct4c01424_si_002.pdf [file ct4c01424_si_002.pdf]

Analyzing Atomic Interactions in Molecules as Learned by Neural Networks

Malte Esders,[*,†,‡] Thomas Schnake,[†,‡,@] Jonas Lederer,[†,‡,@] Adil Kabylda,[¶] Grégoire Montavon,[§,†,‡] Alexandre Tkatchenko,[*,¶] and Klaus-Robert Müller[*,†,‡,‖,⊥,#]

†BIFOLD - Berlin Institute for the Foundations of Learning and Data, 10587 Berlin, Germany

‡Machine Learning Group, Berlin Institute of Technology, 10587 Berlin, Germany

¶Department of Physics and Materials Science, University of Luxembourg, L-1511 Luxembourg City, Luxembourg

§Department of Mathematics and Computer Science, Free University of Berlin, 14195 Berlin, Germany

‖Google Deepmind, 10963 Berlin, Germany

⊥Department of Artificial Intelligence, Korea University, Seoul 136-713, Korea

#Max Planck Institute for Informatics, 66123 Saarbrücken, Germany

@Contributed equally to this work

E-mail: esders@tu-berlin.de; alexandre.tkatchenko@uni.lu; klaus-robert.mueller@tu-berlin.de

S1 Interaction strength, Interaction range and Many-bodyness computation

All explainability results in the main text can be reproduced with algorithm 1.

Data: Graph $\mathcal{G}$; model; walk distance function d
Result: Interaction range, interaction strengths, and many-bodyness

```
// Model forward pass (prediction)
```
$\hat{y} \leftarrow \text{model}(\mathcal{G})$

```
// Relevance backward pass with gnnlrp library based on Schnake et al.
   2022
```
$(\{\mathcal{W}\}, \{\mathcal{R}_\mathcal{W}\}) \leftarrow \text{gnnlrp}(\mathcal{G}, \text{model}, \hat{y})$

for $\mathcal{W} \in \{\mathcal{W}\}$ **do**

$\quad \mathbb{P}_\mathcal{W} \leftarrow \frac{|\mathcal{R}_\mathcal{W}|}{\sum_{\mathcal{W}'} |\mathcal{R}_{\mathcal{W}'}|}$

end

```
// Fourth generalized expectation of the walk distribution
```
$\lambda_4^{\text{pow}} \leftarrow \left[\sum_\mathcal{W} \mathbb{P}_\mathcal{W} d(\mathcal{W}^4) \right]^{\frac{1}{4}}$

```
// Interaction strength computation
```
foreach *atom pair* (i, j) *in atom_pairs*$(\mathcal{G})$ **do**

```
   // Get the set of all walks in which atoms i and j are included
```
$\quad \mathcal{S}_{ij} = \{\mathcal{W} | i \in \mathcal{W} \wedge j \in \mathcal{W}\}$

$\quad s_{ij} \leftarrow \sum_{\mathcal{W} \in \mathcal{S}_{ij}} \mathbb{P}_\mathcal{W}$

end

```
// Many-bodyness computation
// Binning of interaction strengths based on the interatomic distance
```
$\{s\}_R \leftarrow \{s_{ij} | \, ||\boldsymbol{r}_i - \boldsymbol{r}_j|| \approx R\}$

$\{\gamma_R\}_R \leftarrow \log_{10} \left(\frac{P_{100}(\{s\}_R)}{P_{10}(\{s\}_R)} \right)$

$\bar{\gamma} \leftarrow \frac{1}{N_{\text{bins}}} \sum_{\gamma_R \in \{\gamma_R\}_R} \gamma_R$

return *Interaction range* λ*, Many-bodyness* γ

Algorithm 1: Algorithm for computing interaction range, interaction strength, and many-bodyness

S2 Details on the considered GNNs and corresponding relevance attribution

In SchNet,[1,2] the message function between node i and node j is defined as

$$\mu_{ij}^{\mathrm{s}} = \phi(\mathbf{h}_j^{\mathrm{s}}) \circ \mathcal{W}(r_{ij}) \ , \tag{S1}$$

where ϕ is a feed-forward neural network, $\mathbf{h}_i^{\mathrm{s}} \in \mathbb{R}^{D\times 1}$ is the feature representation of node i with the feature dimension D, $\mathcal{W}(r_{ij})$ denotes the continuous convolution filter depending on the pairwise distance r_{ij}, and "$\circ$" denotes the Hadamard (element-wise) product. The combine step is simply

$$\mathcal{C}_i^{\mathrm{s}} = \mathbf{h}_i^{\mathrm{s}} + \sum_{j\in\mathcal{N}(i)} \mu_{ij}^{\mathrm{s}} \ . \tag{S2}$$

PaiNN[3] utilizes vectorial features $\mathbf{h}_i^{\mathrm{v}} \in \mathbb{R}^{D\times 3}$ in addition to the scalar features $\mathbf{h}_i^{\mathrm{s}}$ described above. The message function for the scalar features is equivalent to SchNet (cf. S1), and the message function of the vectorial features is given by

$$\mu_{ij}^{v} = \mathbf{h}_j^{\mathrm{v}} \circ \phi_{\mathrm{vv}}(\mathbf{h}_j^{\mathrm{s}}) \circ \mathcal{W}_{\mathrm{vv}}(r_{ij}) + \phi_{\mathrm{vs}}(\mathbf{h}_j^{\mathrm{s}}) \circ \mathcal{W}'_{\mathrm{vs}}(r_{ij})\hat{\mathbf{r}}_{ij} \ , \tag{S3}$$

where ϕ_{vs} and ϕ_{vv} denote non-linear maps.[3] The equivariant filter $\mathcal{W}'_{vs}(r_{ij})\hat{\mathbf{r}}_{ij}$ is given by the direction of the normalized position difference $\hat{\mathbf{r}}_{ij} = (\mathbf{r}_j - \mathbf{r}_i)/r_{ij}$ scaled by an invariant filter $\mathcal{W}'_{vs}(r_{ij})$. In PaiNN, the combine step is divided into two phases. First, the scalar and the vectorial features are updated individually

$$\mathbf{m}_i^{\mathrm{s}} = \mathbf{h}_i^{\mathrm{s}} + \sum_{j\in\mathcal{N}(i)} \mu_{ij}^{\mathrm{v}} \tag{S4}$$

$$\mathbf{m}_i^{\mathrm{v}} = \mathbf{h}_i^{\mathrm{v}} + \sum_{j\in\mathcal{N}(i)} \mu_{ij}^{\mathrm{v}} \ , \tag{S5}$$

similarly to the residual update in SchNet (cf. S2) and, subsequently, the scalar and vectorial

features are mixed according to

$$\mathcal{C}_i^{\mathrm{s}} = \mathbf{h}_i^{\mathrm{s}} + a_{\mathrm{ss}}(\mathbf{m}_i^{\mathrm{s}}, \|\mathbf{V}\mathbf{m}_i^{\mathrm{v}}\|) + a_{\mathrm{sv}}(\mathbf{m}_i^{\mathrm{s}}, \|\mathbf{V}\mathbf{m}_i^{\mathrm{v}}\|)\langle \mathbf{U}\mathbf{m}_i^{\mathrm{v}}, \mathbf{V}\mathbf{m}_i^{\mathrm{v}} \rangle \ , \tag{S6}$$

$$\mathcal{C}_i^{\mathrm{v}} = \mathbf{h}_i^{\mathrm{v}} + a_{\mathrm{vv}}(\mathbf{m}_i^{\mathrm{s}}, \|\mathbf{V}\mathbf{m}_i^{\mathrm{v}}\|)\mathbf{U}\mathbf{m}_i^{\mathrm{v}} \ . \tag{S7}$$

The functions a_{ss}, a_{sv}, and a_{vv} describe a mixing of non-linearly scaled scalar features s_i and the linearly scaled vectorial features based on the element-wise product and the element-wise sum. $\mathbf{V}$ and $\mathbf{U}$ are linear maps.

While the scalar features are embedded based on the atomic numbers of the respective atom, the vector-features are initialized as $\mathbf{0}$-vectors. We explain the prediction of all architectures with respect to the scalar atomic features. The technical reason for this is that equivariant features are embedded as 0-vectors, which would result in zero relevance attributions. For scalar predictions, such as energy predictions, explaining with respect to scalar features is particularly justified since the predictions solely rely on the final scalar features. The relevance attributions are efficiently computed by exploiting automatic differentiation based on backpropagation.[4] To exclude the vectorial features from the relevance attribution, we detach them in the combine step from the computation graph of the backpropagation. This way the gradient is solely computed for the scalar features, and consequently all relevance is attributed to the scalar features.

S3 Number of walks decreases exponentially

Since our definition of the atom interaction strength involves a sum over walk relevances, the number of walks plays a central role for the interaction strength. The number of walks between two atoms can be approximated in terms of their distance and the number of interaction layers of the GNN.

We denote with $|\mathcal{S}_{ij}|$ the number of walks between atoms i and j. It can be approximated

as:

$$|\mathcal{S}_{ij}| = \begin{cases} \left(\frac{4\pi}{3}c^3\rho\right)^L & \text{if } d \leq c \\ \left(\frac{4\pi}{3}c^3\rho\right)^{(L-\frac{d}{c})} & \text{if } c < d \leq Lc \\ 0 & \text{if } d > Lc \end{cases} \tag{S8}$$

where d is the distance between two atoms, c is the cutoff length, L is the number of message passing layers and ρ is the density of atoms. The term $\frac{4\pi}{3}c^3\rho$ can be thought of as the average number of neighbors within the cutoff distance.

One can see that there is a discontinuity at the cutoff distance. The discontinuity can be explained by the fact that above the cutoff, at least two "hops" are needed to connect two atoms, which abruptly constrains the total number of possible walks between two atoms. Interestingly, when considering the atom interaction strengths, this discontinuity is barely visible anymore (see Figure 5). This smoothing out of the discontinuity is due to the cosine cutoff of the GNN filters.

The predicted number based on this formula aligns qualitatively with the empirically found

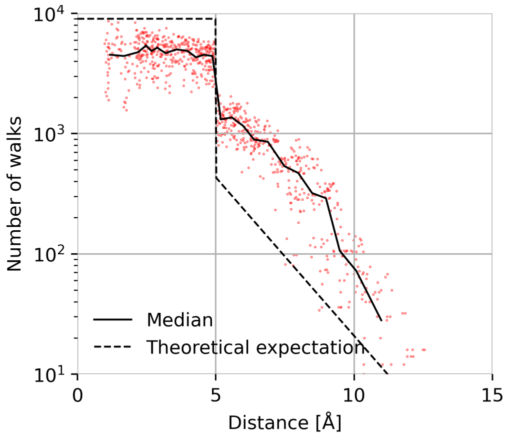

Figure S1. Theoretically expected and empirically found number of walks for each pair of atoms. The GNN is a PaiNN with three interaction layers and a cutoff of 5 Å. The molecule is Ac-Ala3-NHMe. Each point represents an atom pair.

number of walks (Figure S1). Importantly, the slope of the prediction and the empirical median line are similar. This shows that both theoretically and empirically, the number of walks decreases expentially with the interatomic distance. The relationship is fundamentally

the same for all GNNs with a cutoff and indicates an inductive bias of this architecture.

S4 Further results statistics

Here, we show the full results table, an extension of Table 1 from the main text. In the full table, we sometimes evaluate the statistics on a different molecule than what we trained on. This is to make the results more comparable: except for the QM9 results, we always evaluate on the Ac-Ala3-NHMe molecule. In some cases, we want to find out the effect of training on different molecules, and train on the molecule indicated in the table row. For a discussion of training on various other molecules, see S5.

S5 Influence of training data on interaction range

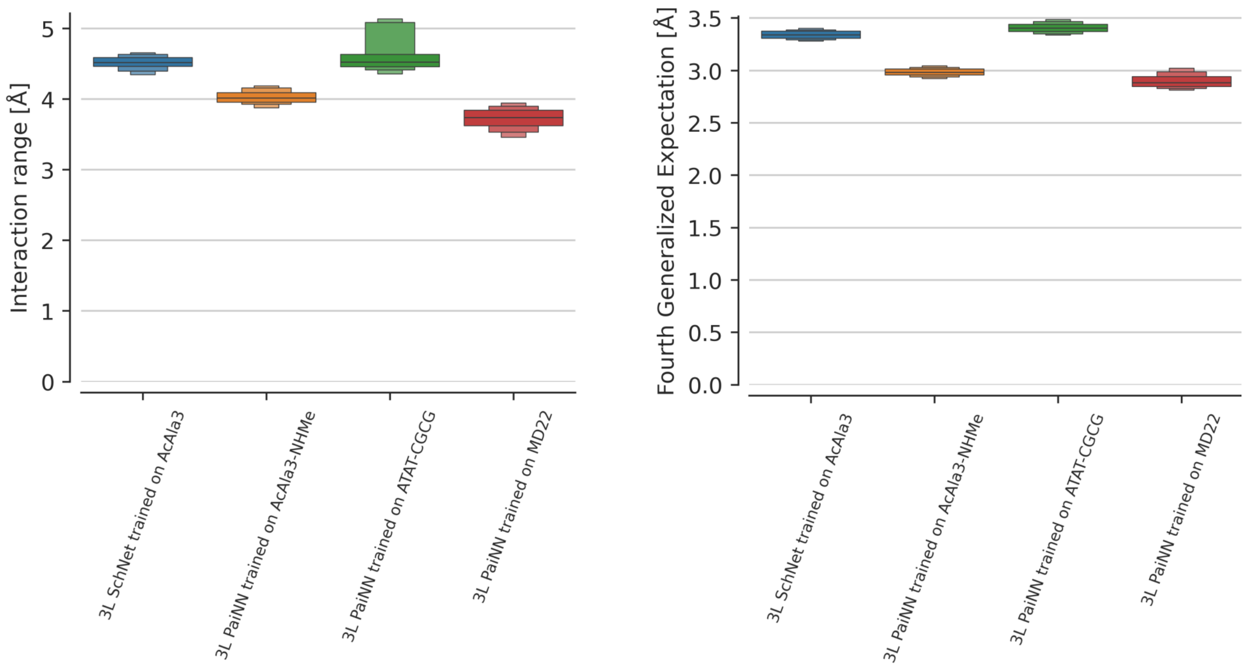

Figure S2. Interaction range for various networks. To make the results comparable, the interaction ranges have been evaluated on Ac-Ala3-NHMe in each case, regardless of the molecule they were trained on. **Left**: Interaction range evaluated with the threshold-measure, eq 8. **Right**: Interaction range evaluated with the fourth generalized expectation, eq 9.

Table S1. Interaction range and many-bodyness statistics. For the experiments with QM9 data, networks were trained and evaluated on the indicated property. For the experiments with Ac-Ala3-NHMe, networks were trained on energies and forces, and evaluated on the energies. For the interaction range, the thresholded range (eq 8) with $p_{\mathrm{min}} = 0.001$ is displayed, and additionally the first and fourth generalized expectation of the walk-length distribution (eq 9 with $a = 1$ and $a = 4$). The many-bodyness has been evaluated with eq 13. The row where the training data is called "MD22 (exc. tubes)" is for a PaiNN that has been trained on all molecules in the MD22 dataset (except the nanotubes).

			Interaction range measures			
Model	Property	Data	$\lambda^{\mathrm{thresh}}_{0.001}$ (eq 8)	$\lambda^{\mathrm{pow}}_{1}$ (eq 9)	$\lambda^{\mathrm{pow}}_{4}$ (eq. 9)	Many-bodyness $\bar{\gamma}$ (eq 13)
3L SchNet	Energy	QM9	4.14	1.62	2.75	0.85
3L SchNet	Dipole	QM9	6.34	2.64	3.37	1.40
3L SchNet	HOMO	QM9	7.04	3.10	3.93	0.87
3L SchNet	LUMO	QM9	7.01	3.03	3.76	0.95
3L PaiNN	Energy	QM9	3.88	1.64	2.44	0.92
3L PaiNN	Dipole	QM9	4.18	1.68	2.70	1.10
3L PaiNN	HOMO	QM9	6.97	2.56	3.43	0.93
3L PaiNN	LUMO	QM9	6.96	2.63	3.56	1.10
1L PaiNN	Energy	Ac-Ala3-NHMe	8.55	2.63	4.41	0.70
2L PaiNN	Energy	Ac-Ala3-NHMe	5.57	2.18	3.17	0.54
3L PaiNN	Energy	Ac-Ala3-NHMe	4.03	2.26	2.98	0.80
4L PaiNN	Energy	Ac-Ala3-NHMe	2.92	1.79	2.50	1.00
5L PaiNN	Energy	Ac-Ala3-NHMe	2.57	1.71	2.29	1.70
1L PaiNN	Energy	Ac-Ala3-NHMe 1k	8.55	2.40	4.43	0.63
2L PaiNN	Energy	Ac-Ala3-NHMe 1k	5.89	2.03	3.30	0.45
3L PaiNN	Energy	Ac-Ala3-NHMe 1k	3.59	1.74	2.67	0.76
4L PaiNN	Energy	Ac-Ala3-NHMe 1k	2.79	1.54	2.28	1.10
5L PaiNN	Energy	Ac-Ala3-NHMe 1k	2.48	1.37	1.96	1.90
1L PaiNN untrained	Energy	Ac-Ala3-NHMe	8.55	1.15	3.69	0.46
2L PaiNN untrained	Energy	Ac-Ala3-NHMe	4.68	1.05	2.99	0.40
3L PaiNN untrained	Energy	Ac-Ala3-NHMe	2.60	0.52	1.89	0.78
4L PaiNN untrained	Energy	Ac-Ala3-NHMe	1.94	0.20	1.27	1.30
5L PaiNN untrained	Energy	Ac-Ala3-NHMe	1.63	0.087	0.88	2.30
3L SchNet	Energy	Ac-Ala3-NHMe	4.54	2.62	3.34	0.72
3L PaiNN	Energy	ATAT-CGCG	4.61	2.60	3.41	0.76
3L PaiNN	Energy	MD22 (exc. tubes)	3.73	1.95	2.90	0.76

We trained 3L PaiNNs on Ac-Ala3-NHMe, ATAT-CGCG (also from MD22) and on all molecules in MD22 combined (except the nanotubes) to find out whether the type of molecule used for training has an effect on the interaction range. We also compare to a SchNet trained on Ac-Ala3-NHMe. In the direct comparison between SchNet and PaiNN, PaiNN seems to have a slightly lower range (Figure S2), which is a trend that we already observed on the QM9 data for the energy property, particularly in the fourth generalized expectation measure (Table S1). Comparing the three PaiNNs trained on Ac-Ala3-NHMe, ATAT-CGCG and all molecules in MD22 shows that the model trained on all molecules has a lower interaction range, whereas the one trained on ATAT-CGCG has a slightly higher range. We speculate that fitting the long-distance interactions on all molecules at the same time is a too difficult learning task because the long-distance interactions are heavily dependent on the type of molecule. The short-range interactions on the other hand are more similar between molecules and are also more important factors for predicting the energy/forces than the long range interactions. Therefore, training focuses mostly on the short-range interactions, which leads to a lower interaction range. On the other hand, ATAT-CGCG is a larger system than Ac-Ala3-NHMe, comprised of four molecules with long-range inter-molecular forces. Here, in line with this fact, the interaction range is the largest.

S5.1 Results of explaining on larger molecules

To investigate the learned representations on significantly larger molecules, we have evaluated the explainability method also on the molecules DHA (56 atoms) and buckyball-catcher (148 atoms) from the MD22 dataset. Note that these results are different from those in table S1, because the results in the table are all evaluated on Ac-Ala3-NHMe, irrespective of the molecule the models were trained on. The results discussed in this paragraph are for models trained and evaluated on DHA and Buckyball Catcher. Results for these are shown in Fig. S3. What we see is that two of the principles we identified in the main text, namely the

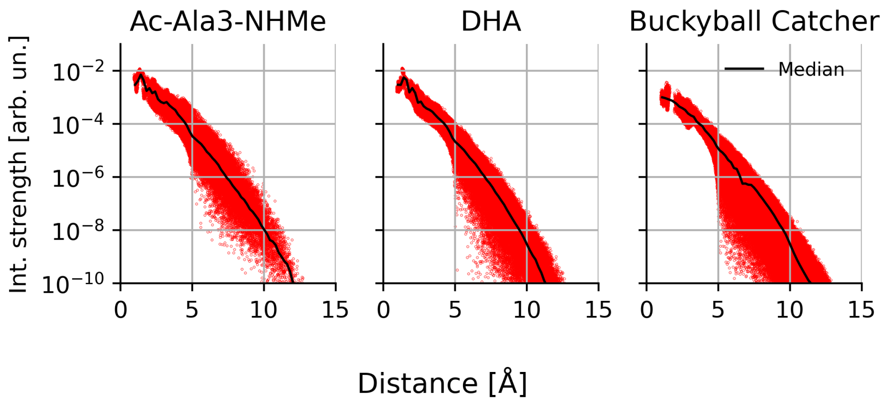

Figure S3. Interaction strength decay across inter-atomic distance for three molecules. The GNN in each case was a 3-layer PaiNN with a 5 Å cutoff. **Left**: Ac-Ala3-NHMe (42 atoms); **Middle**: Docosahexaenoic acid (DHA, 56 atoms); **Right**: Buckyball Catcher (148 atoms).

exponential (rather than polynomial) decay of the interaction strength, and the increase of the many-bodyness with distance, are present in DHA and the Buckyball Catcher as well. What is striking is that the effect of the GNN single-layer cutoff, at 5 Å, is visible in all three molecules, but is much more pronounced in the Buckyball Catcher. The reason could be the distance of the "ball" to the "catcher", which makes atom pairs where one atom is in the ball and the other in the catcher interact weakly, whereas atom pairs within either the ball or the catcher interact stronger.

S6 Computational details: GNN Training and MD

All results throughout the paper were computed according to algorithm 1. The models were trained using SchNetPack,[5,6] using the parameters in Table S2.

S7 Analysis of models overfitting to a restricted training set

Overfitting is a ubiquitous problem in machine learning. Therefore, we want to see the effect of overfitting on our explainability analysis. We hypothesize that models that adhere to

Table S2. Parameters used for training. For the 1-5 layer experiments, some parameters were adapted as in Table 2.

Parameter	Value
Batch size	100
Weight decay	0.01
Learning rate	0.0005
N interaction layers	3
Atomic feature embedding	128
Radial basis function	Gaussian
N radial basis functions	20
Cutoff function	Cosine
Cutoff length [Å]	5

known physico-chemical principles will be less prone to overfitting. To test this, we subsequently perform MD stability analysis as described in Section 3.3.

We once again take Ac-Ala3-NHMe as our test-system. To induce overfitting and create a somewhat realistic test-case, we selected only 1k training points (as opposed to 72k training points as for the models in the main text), and we sample the training points only from a small region of the configuration space. To do this, we project all data onto two representative peptide-bond angles ϕ and ψ. As can be seen in Fig. S4, most samples in the original dataset fall into a minimum around $\phi = -1.5, \psi = 0$ radians. To construct a new training set, we sampled points only from the region $-1 < \psi < 0.5$, i.e. around the largest minimum (see Fig. S4).

Models fitted on this reduced training set have a significantly more challenging extrapolation task, and the low number of training points also makes overfitting more likely.

We once again trained the 1-5-layer PaiNNs described in the paper in this restricted data setting. We then again ran 30 MD trajectories per network and observed how many trajectories were unstable (Table S3). As expected, the MD stability of the models trained on the reduced set was worse in almost all cases. The only exception is the 1-layer network: When it was trained on the full dataset, it was very unstable while having a very low validation error, but when trained on the reduced dataset it was perfectly stable (0/30 instable trajectories)

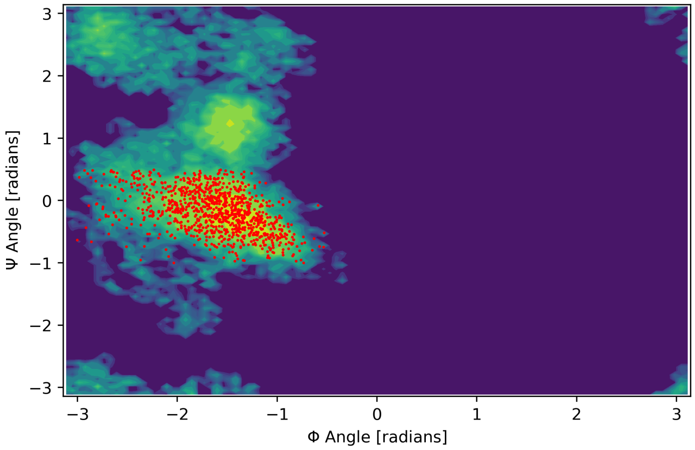

Figure S4. Projection of configurations of Ac-Ala3-NHMe onto two dihedral peptide-bond angles ψ and ϕ. The background color indicates the likelihood of occurence of each state as seen in the MD22 dataset. The red dots indicate the sampled training points.

while having a high validation error. This curious result highlights the fact that neither the validation accuracy nor the MD stability alone are good measures of the generalization performance.

Regarding the 2-5-layer models, the results were in line with our hypothesis that models that are in accordance with expected physico-chemical principles generalize better: While all models trained on the reduced dataset had significantly worse validation error, the two models that were in line with chemical principles III and IV, namely the 2- and 3-layer networks, still produced almost perfectly stable MD trajectories. This is remarkable given the fact that much of the MD trajectory they perform is in regions of conformational space from which they had not "seen" any training data.

Evaluating the interaction strengths for these models and comparing them to those of the models trained on the unconstrained training set (Fig. S4), it may look at first sight as if they are extremely similar. However, keep in mind that the y-axis is logarithmic. One interesting aspect is that for the 2-5-layer networks, the many-bodyness at lower interatomic distance is lower for the models trained with the restricted set than for those trained on the full training set. The interaction strength of the 1-layer model decreases sharply towards the end of the interatomic distance, which is in stark contrast to the 1-layer model trained on

Table S3. Test accuracies and MD instability for versions of PaiNN with various amounts of interaction layers. Additionally, variants of PaiNN that were trained on a severely reduced training set, see text in Section S7. The cutoffs are measured in Å, energy in kcal mol^{-1}, and forces in kcal/mol/Å. RMSE: Root Mean Squared Error, MAE: Mean Absolute Error.

Model	N Train data	Cutoff	Property	RMSE	MAE	MD failures
1L PaiNN	72k	15	Energy	0.13	0.10	22/30
			Forces	0.25	0.19	
2L PaiNN	72k	7.5	Energy	0.17	0.13	0/30
			Forces	0.25	0.18	
3L PaiNN	72k	5	Energy	0.11	0.09	0/30
			Forces	0.14	0.10	
4L PaiNN	72k	3.75	Energy	0.28	0.22	4/30
			Forces	0.27	0.19	
5L PaiNN	72k	3	Energy	0.41	0.33	11/30
			Forces	0.33	0.24	
1L PaiNN	1k	15	Energy	1.54	1.06	0/30
			Forces	2.14	1.52	
2L PaiNN	1k	7.5	Energy	0.92	0.59	0/30
			Forces	1.10	0.71	
3L PaiNN	1k	5	Energy	0.70	0.46	2/30
			Forces	0.90	0.58	
4L PaiNN	1k	3.75	Energy	0.58	0.42	8/30
			Forces	0.94	0.59	
5L PaiNN	1k	3	Energy	0.95	0.69	22/30
			Forces	1.11	0.72	

the full set.

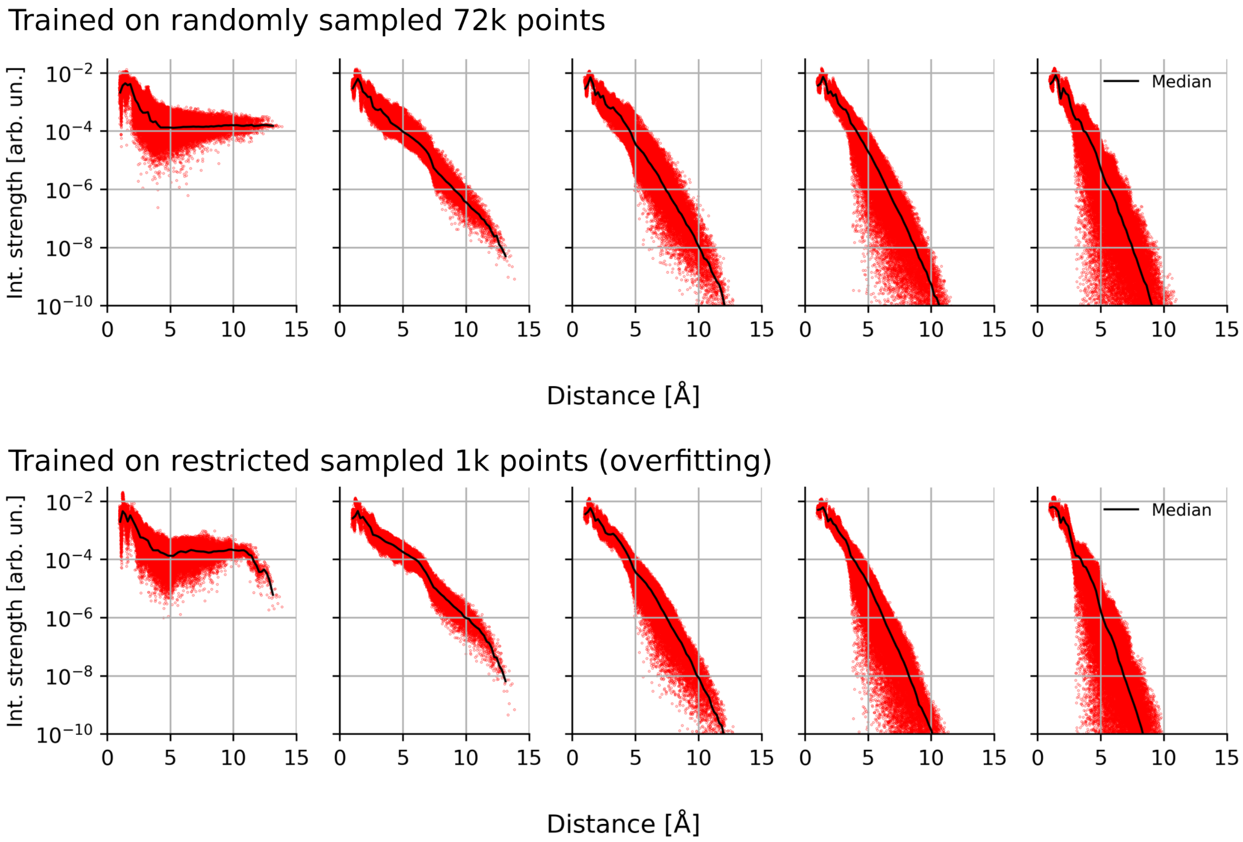

Figure S5. Interaction strength decay of 1-5-layer PaiNNs (left to right) on Ac-Ala3-NHMe. Upper row: The training set consisted of 72k randomly sampled points, like in the results in the main text. Lower row: The training set consisted of 1k samples sampled from a restricted region of configurational space (see Fig. S4).

References

(1) Schütt, K. T.; Sauceda, H. E.; Kindermans, P.-J.; Tkatchenko, A.; Müller, K.-R. Schnet– a deep learning architecture for molecules and materials. *The Journal of Chemical Physics* **2018**, *148*.

(2) Schütt, K. T.; Kindermans, P.-J.; Felix, H. E. S.; Chmiela, S.; Tkatchenko, A.; Müller, K.-R. SchNet: A continuous-filter convolutional neural network for modeling quantum interactions. Neural Information Processing Systems. 2017; pp 991–1001.

(3) Schütt, K. T.; Unke, O.; Gastegger, M. Equivariant message passing for the prediction of tensorial properties and molecular spectra. International Conference on Machine Learning. 2021; pp 9377–9388.

(4) Montavon, G.; Binder, A.; Lapuschkin, S.; Samek, W.; Müller, K.-R. *Explainable AI*; Lecture Notes in Computer Science; Springer, 2019; Vol. 11700; pp 193–209.

(5) Schütt, K. T.; Kessel, P.; Gastegger, M.; Nicoli, K. A.; Tkatchenko, A.; Müller, K.-R. SchNetPack: A Deep Learning Toolbox For Atomistic Systems. *Journal of Chemical Theory and Computation* **2019**, *15*, 448–455.

(6) Schütt, K. T.; Hessmann, S. S. P.; Gebauer, N. W. A.; Lederer, J.; Gastegger, M. SchNetPack 2.0: A neural network toolbox for atomistic machine learning. *The Journal of Chemical Physics* **2023**, *158*, 144801.
